# Supplementary material for: Psychological stress impairs IL22-driven protective gut mucosal immunity against colonising pathobionts
Source: Nat Commun. 2021 Nov 18;12:6664. doi: 10.1038/s41467-021-26992-4 (PMC8602651; doi:10.1038/s41467-021-26992-4)
Supplement: Supplementary file 1 — Supplementary Information [file 41467_2021_26992_MOESM1_ESM.pdf]

Supplementary Information for:

Psychological stress impairs IL22-driven protective gut mucosal immunity against colonising pathobionts

Christopher R. Shaler [1,2,+], Alexandra A. Parco [1,2,+], Wael Elhenawy [1,2], Jasmeen Dourka [1,2], Jennifer Jury [3], Elena F. Verdu [3], Brian K. Coombes [1,2,3,*]

[1] Department of Biochemistry and Biomedical Sciences, McMaster University, Hamilton, ON, Canada; [2] Michael G. DeGroote Institute for Infectious Disease Research, Hamilton, ON, Canada;
[3] Farncombe Family Digestive Health Research Institute, Hamilton, ON, Canada

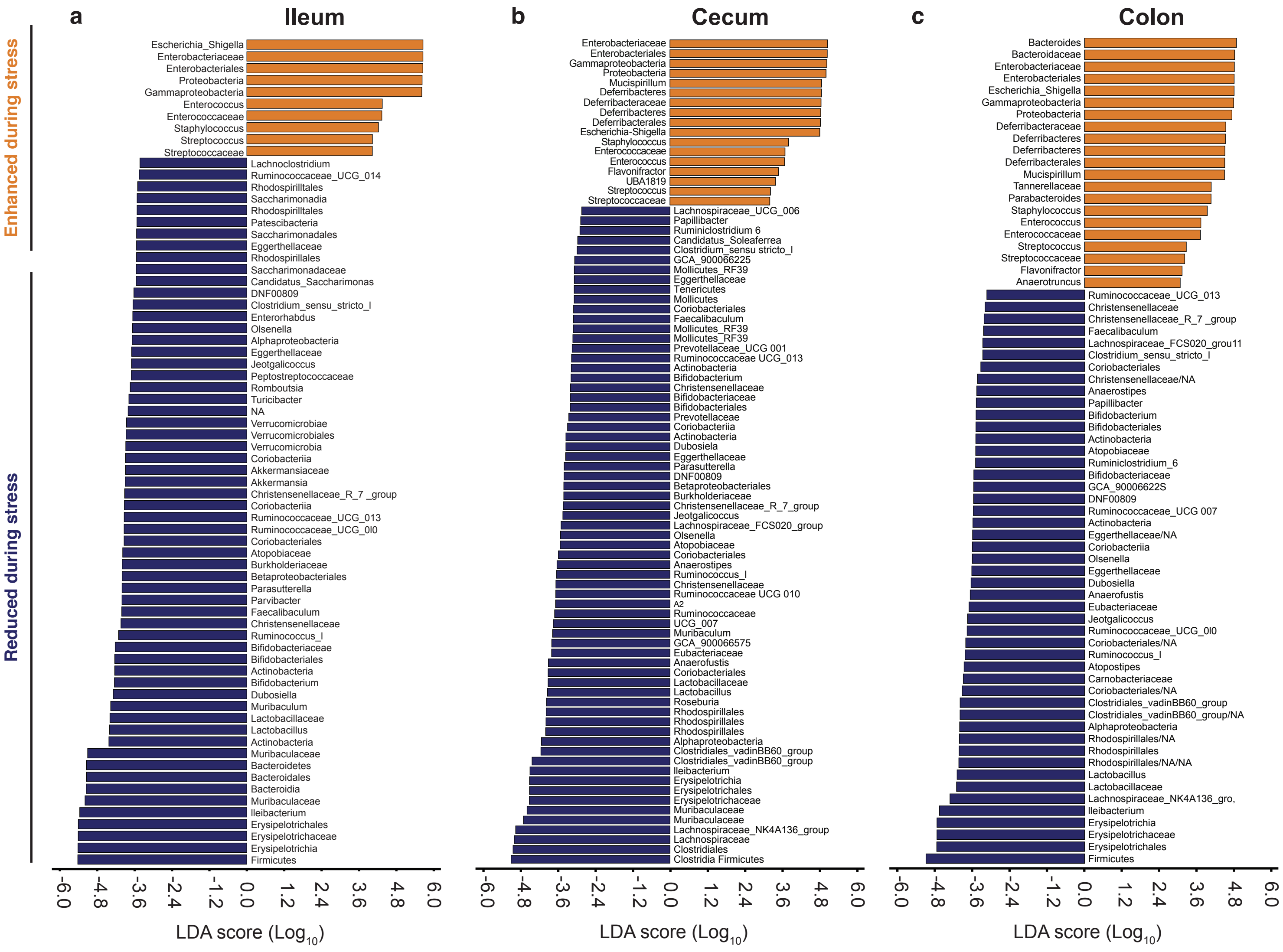

Supplementary Figure 1. Psychological stress alters the microbial composition of the gut. LEfSe analysis of significantly altered bacterial populations in the ileum (**a**), cecum (**b**), and colon (**c**) of stressed mice (n = 4) as compared to starved controls (n = 4). Starved and stress samples are the same as those in Figure 1.

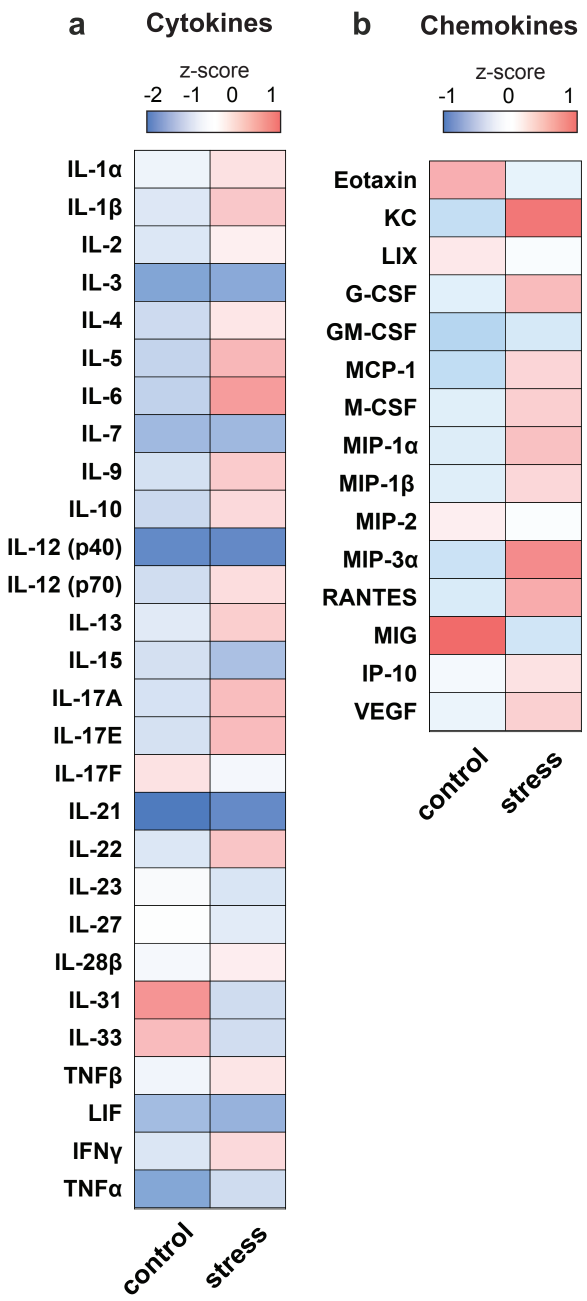

Supplementary Figure 2. Psychological stress induces the release of pro-inflammatory cytokines within the serum. **a**, Mean z-scores of cytokine levels within the serum of control (n = 6) and stress (n = 7) mice as determined by Multiplex analysis. **b**, Mean z-scores of chemokine levels within the serum of control (n = 6) and stress (n = 7) mice as determined by Multiplex analysis.

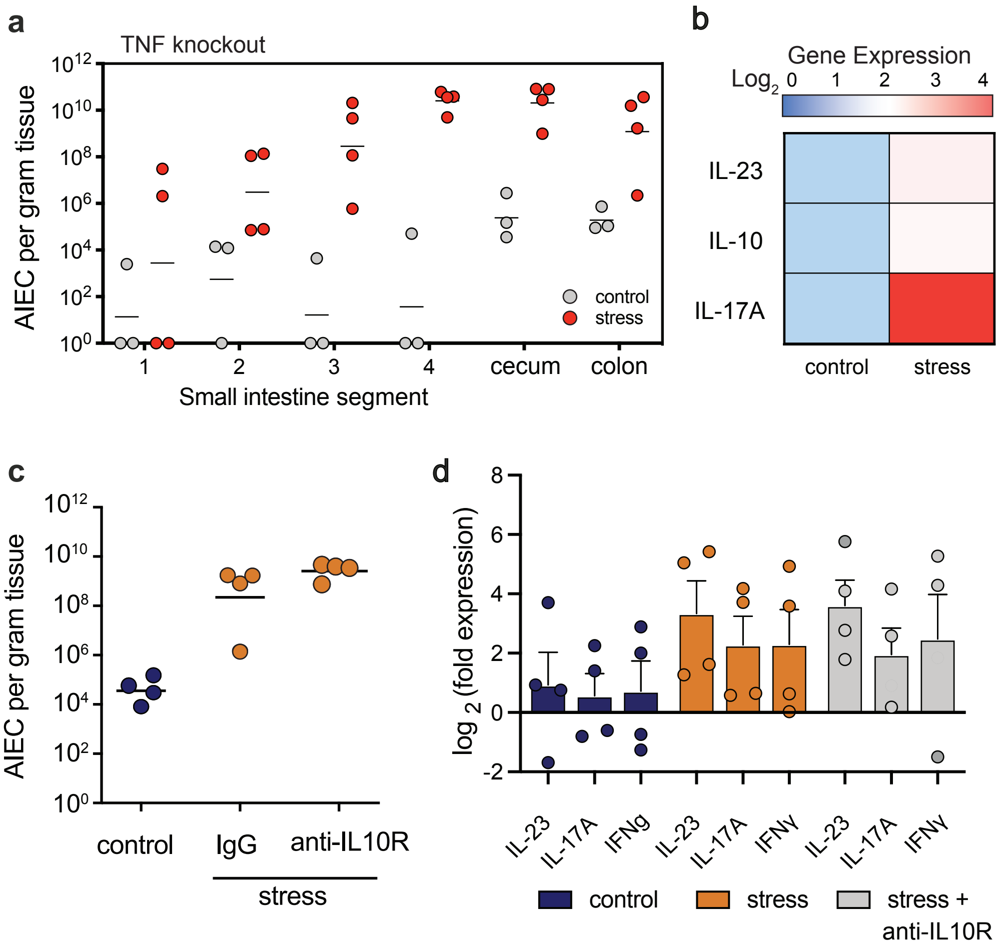

Supplementary Figure 3. TNF does not contribute to stress induced AIEC expansion. **a**, AIEC tissue burdens collected from the length of the intestinal tract from control (n = 3) and stress (n = 4) TNFko mice. The small intestine was divided into four 8 cm segments in which Segment 4 is adjacent to the cecum. **b**, RT-qPCR analysis of cytokine expression within the ileum of control (n = 2) and stress (n = 4) TNFKO mice. **c**, AIEC tissue burdens collected from the ileum of control (n = 4), stress + IgG (n = 4), and stress + IL-10R (n = 4). **d**, RT-qPCR analysis of cytokine expression within the ileum of control (n = 4), stress + IgG (n = 4), and stress + anti-IL-10R (n = 4). Error bars represent SEM and the line in CFU graphs indicates the geometric mean of the group.

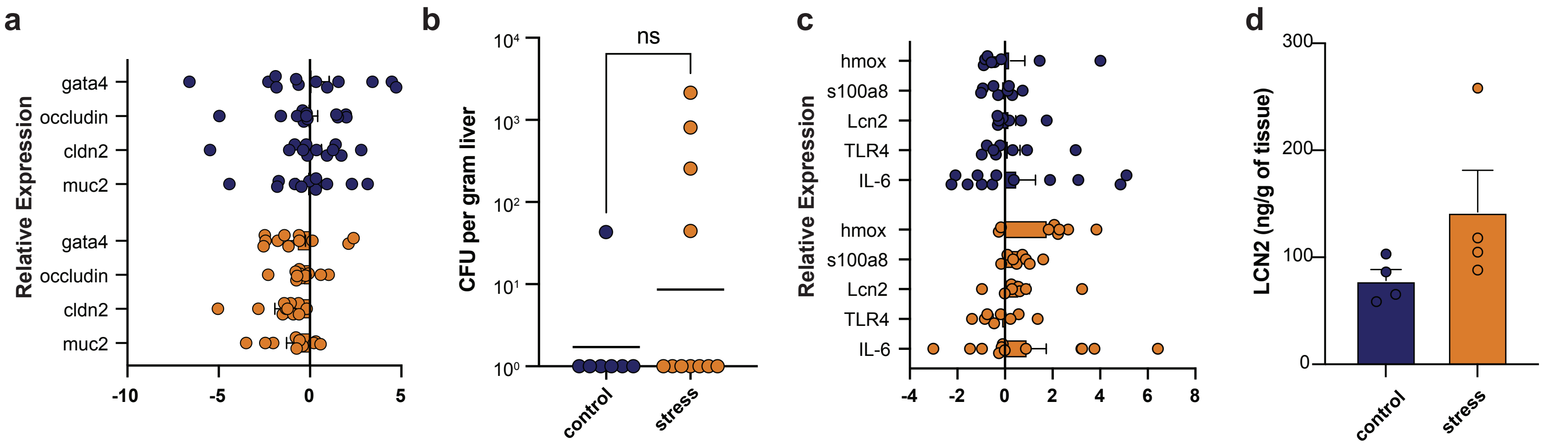

Supplementary Figure 4. Psychological stress leads to modest impairment of barrier function and induction of nutritional immunity in naïve mice. **a**, RT-qPCR analysis of barrier genes from ileal samples of naïve starved (n = 12) and stress (n = 11) mice. **b**, Bacterial burdens in the liver were enumerated in naïve starved (n = 12) or stressed (n = 12) mice. Significance was determined by a two-sided Mann-Whitney. **c**, RT-qPCR analysis of nutritional immunity genes from ileal samples of naïve starved (n = 8) and stress (n = 8) mice. **d**, Quantification of Lcn2 by ELISA in the ileum of naïve starved (n = 4) and stress (n = 4) mice. Error bars represent SEM and the line in CFU graphs indicates the geometric mean of the group.

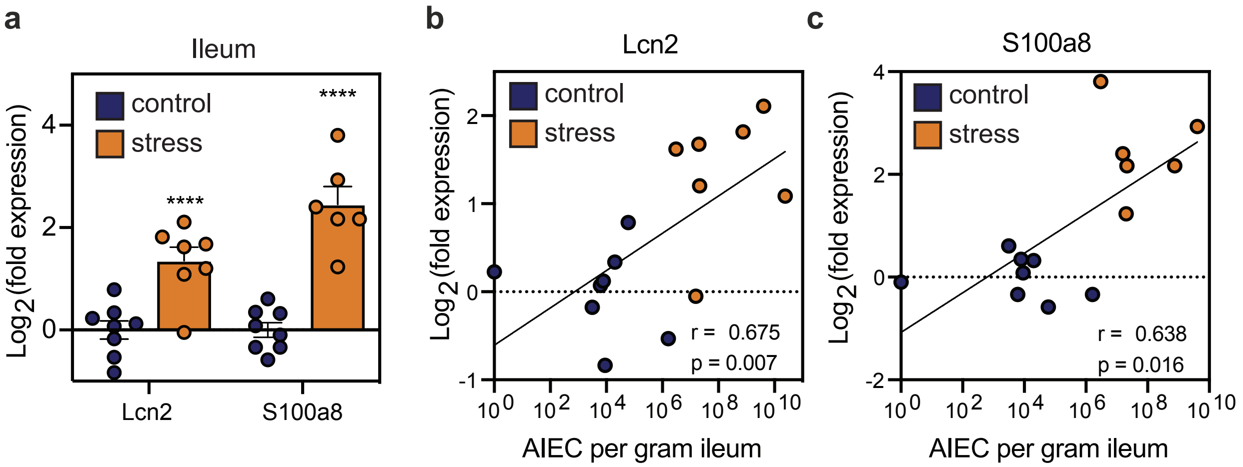

Supplementary Figure 5. Psychological stress induces nutritional immunity. **a**, RT-qPCR analysis of Lcn2 and S100a8 from ileal samples of AIEC-colonized starved (n = 4) or stressed (n = 4) mice. Significance was determined by a two-way ANOVA, $p < 0.0001$ (Lcn2); $p < 0.0001$ (S100a8). **b**, Spearman's two tailed correlation of RT-qPCR Lcn2 transcript expression and AIEC fecal burdens within the ileum (n = 8). **c**, Spear-man's two tailed correlation of RT-qPCR S100a8 transcript expression and AIEC fecal burdens within the ileum (n = 8). (* $p \leq 0.05$; ** $p \leq 0.01$; *** $p \leq 0.001$; **** $p \leq 0.0001$). Error bars represent SEM.

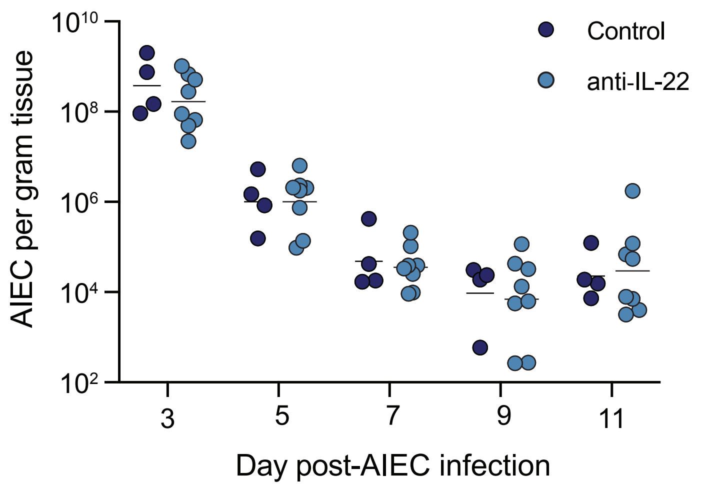

Supplementary Figure 6. IL-22 neutralization alone does not cause AIEC expansion. Fecal AIEC burdens collected from AIEC-colonized control (n = 4) and IL-22 treated (n = 8) over the course of 11 days.

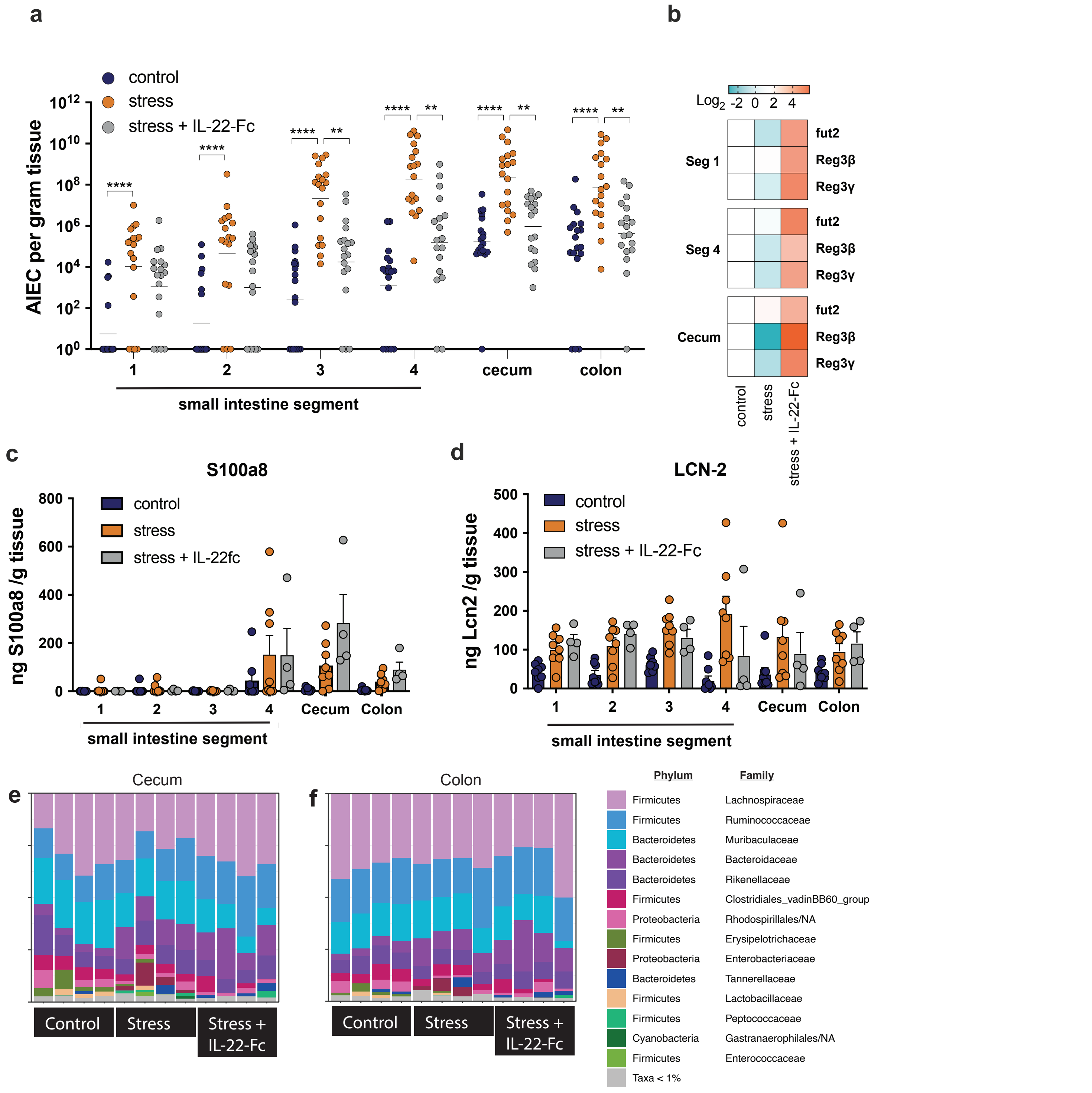

Supplementary Figure 7. IL-22 induces the production of AMPs and limits AIEC expansion.
a, AIEC tissue burdens collected from the length of the intestinal tract from control (n = 18), stress (n = 18), and IL-22 treated stress (n = 18) mice. The small intestine was divided into four 8 cm segments in which Segment 4 is adjacent to the cecum. Significance was determined by one-way ANOVA, within each segment. Segment 1 - Control:stress p<0.0001; Segment 2 - Control:Stress p< 0.0001, Stress:Stress + IL-22-Fc p=0.023; Segment 3 - Control:Stress p<0.0001, Stress:Stress + IL-22-Fc p = 0.0012; Segment 4 - Control:Stress p<0.001, Stress:Stress + IL-22-Fc p = 0.001; Cecum - Control:Stress p<0.0001, Stress:Stress + IL-22-Fc p = 0.0006; Colon - Control:Stress p<0.0001, Stress:Stress+IL-22-Fc p=0.0021 **b**, RT-qPCR analysis of AMP expression in ileal samples of AIEC-colonized starved (n = 4), IgG treated stress (n = 4), and IL-22 treated stress (n = 4) mice. Stress and control samples are the same as Figure 4B. **c**, Quantification of calprotectin subunit S100a8 by ELISA along the length of the intestinal tract in AIEC-colonized starved (n = 8), stress (n = 8), and IL-22 treated stress (n = 4) mice. Control and stress samples are the same as Figure 4D. **d**, Quantification of Lcn2 by ELISA along the length of the intestinal tract in AIEC-colonized starved (n = 8), stress (n = 8), and IL-22 treated stress (n = 4) mice. Control and stress samples are the same as Figure 4C. **e, f,** Taxonomy plots of 16S rRNA sequencing of the cecal e and colonic f contents of starved (n = 4), IgG treated stress (n = 4), and IL-22 treated stress (n = 4) mice. The small intestine was divided into four 8 cm segments in which Segment 4 was adjacent to the cecum. (* p ≤ 0.05; ** p ≤ 0.01; *** p ≤ 0.001; **** p ≤ 0.0001). Error bars represent SEM and the line in CFU graphs indicates the geometric mean of the group.

a

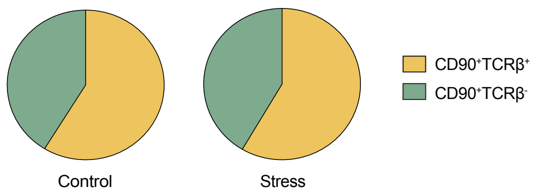

Of CD45⁺	Control	Stress
CD90⁺ TCRβ⁺	58.76% ± 6.7%	58.46% ± 10%
CD90⁺ TCRβ-	41.24% ± 3.4%	41.54% ± 7.3%

b

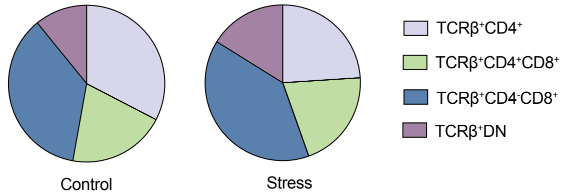

Of TCRβ⁺CD90⁺	Control	Stress
CD4⁺	32.59% ± 5.0%	23.97% ± 4.8%
CD4⁺CD8⁺	20.24% ± 4.4%	20.59% ± 5.9%
CD4⁺CD8⁻	36.28% ± 3.9%	39.28% ± 5.0%
CD4⁻CD8⁻	10.89% ± 1.2%	16.16% ± 2.7%

c

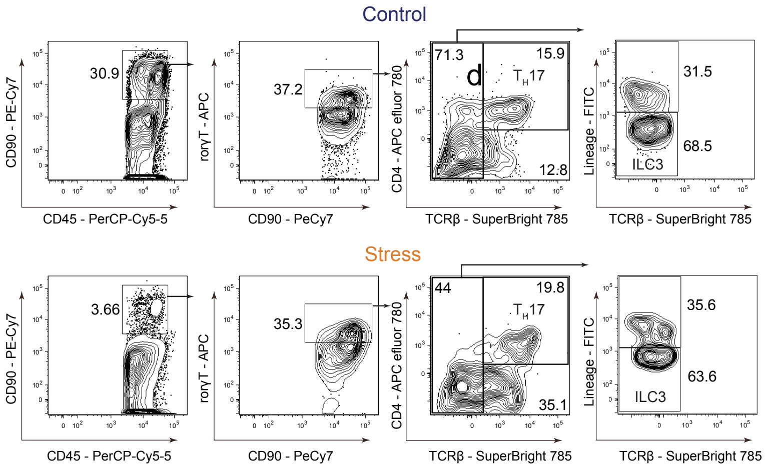

Of CD90⁺	Control	Stress
T_H17	7.4 ± 1.5%	5.4 ± 0.5%
ILC3	6.8 ± 3.8%	4.5 ± 1.4%

Total	Control	Stress
T_H17	$3.1 \times 10^5 \pm 2.2 \times 10^5$	$3.7 \times 10^4 \pm 2.1 \times 10^4$
ILC3	$7.5 \times 10^5 \pm 7.3 \times 10^5$	$2.1 \times 10^4 \pm 9.6 \times 10^3$

Supplementary Figure 8. Psychological stress leads to an unbiased loss of the CD90+ immune population. a, Proportion of CD90+TCRβ+ and CD90+TCRβ- in control (n = 10) and stress (n = 5) mice. **b**, Proportion of CD90+TCRβ+ that are CD4+, CD8+, CD4+CD8+, or CD4-CD8- in control (n = 10) and stress groups (n = 5). **c**, Gating strategy for TH17 and ILC3 from CD45+CD90+ cells.

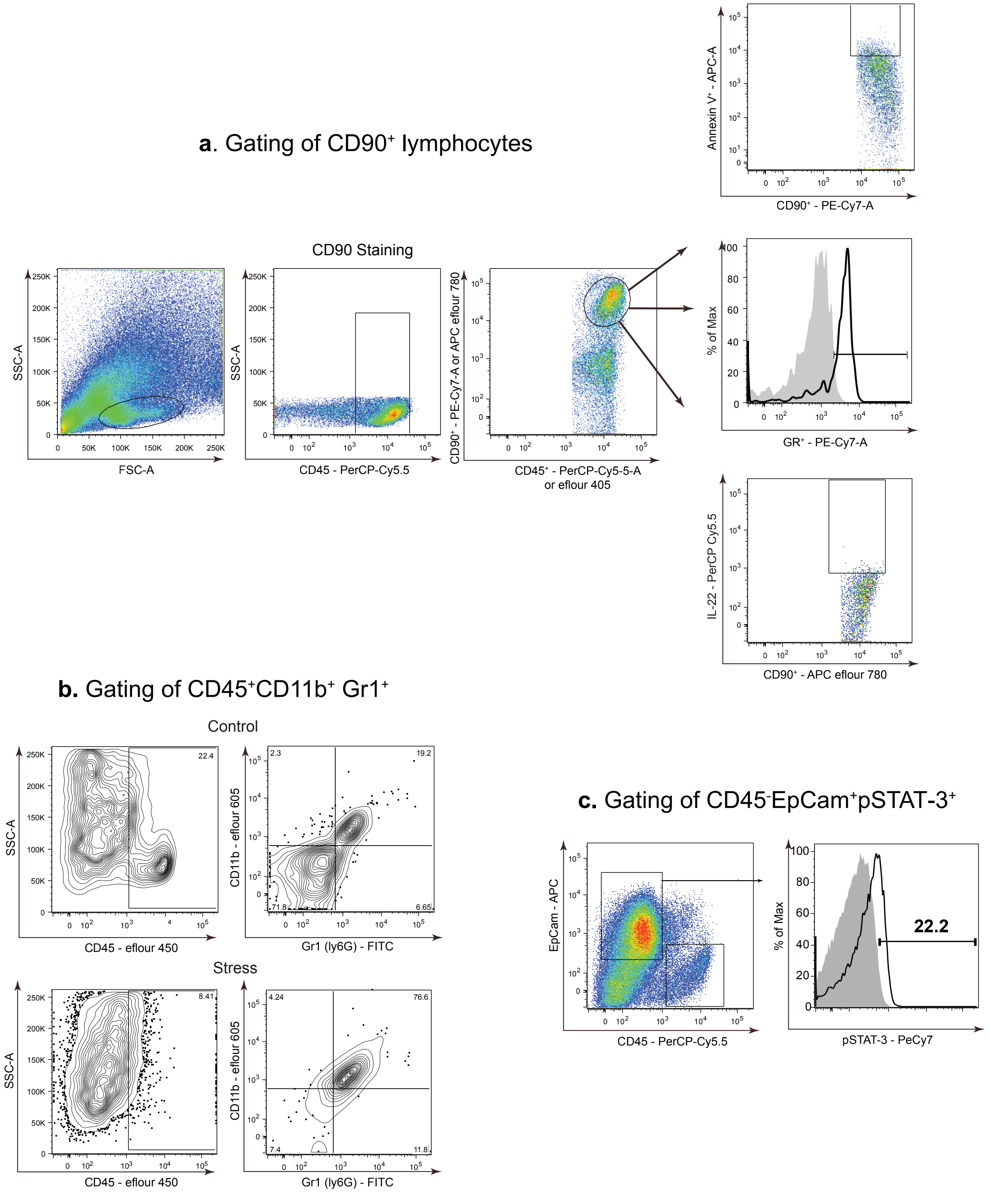

Supplementary Figure 9. FACS gating strategies. a, The gating methods used for defining CD90+ lymphocytes and their expression of IL-22, glucocorticoid receptor, or annexin V. **b**, The gating methods used for CD45+CD11b+Gr1 (ly6G)+ cells. **c**, The gating methods used to determine pSTAT-3 expression on CD45- EpCam+ epithelial cells.
