# Supplementary material for: Psychological stress impairs IL22-driven protective gut mucosal immunity against colonising pathobionts
Source: Nat Commun. 2021 Nov 18;12:6664. doi: 10.1038/s41467-021-26992-4 (PMC8602651; doi:10.1038/s41467-021-26992-4)
Supplement: Supplementary file 3 — Reporting Summary [file 41467_2021_26992_MOESM3_ESM.pdf]

nature research

Corresponding author(s): Brian Coombes

Last updated by author(s): October 12, 2021

Reporting Summary

Nature Research wishes to improve the reproducibility of the work that we publish. This form provides structure for consistency and transparency in reporting. For further information on Nature Research policies, see our Editorial Policies and the Editorial Policy Checklist.

Statistics

For all statistical analyses, confirm that the following items are present in the figure legend, table legend, main text, or Methods section.

n/a	Confirmed	
☐	☒	The exact sample size (*n*) for each experimental group/condition, given as a discrete number and unit of measurement
☐	☒	A statement on whether measurements were taken from distinct samples or whether the same sample was measured repeatedly
☐	☒	The statistical test(s) used AND whether they are one- or two-sided<br>*Only common tests should be described solely by name; describe more complex techniques in the Methods section.*
☐	☒	A description of all covariates tested
☒	☐	A description of any assumptions or corrections, such as tests of normality and adjustment for multiple comparisons
☐	☒	A full description of the statistical parameters including central tendency (e.g. means) or other basic estimates (e.g. regression coefficient) AND variation (e.g. standard deviation) or associated estimates of uncertainty (e.g. confidence intervals)
☐	☒	For null hypothesis testing, the test statistic (e.g. *F*, *t*, *r*) with confidence intervals, effect sizes, degrees of freedom and *P* value noted<br>*Give P values as exact values whenever suitable.*
☒	☐	For Bayesian analysis, information on the choice of priors and Markov chain Monte Carlo settings
☒	☐	For hierarchical and complex designs, identification of the appropriate level for tests and full reporting of outcomes
☐	☒	Estimates of effect sizes (e.g. Cohen's *d*, Pearson's *r*), indicating how they were calculated

Our web collection on statistics for biologists contains articles on many of the points above.

Software and code

Policy information about availability of computer code

Data collection	Lightcyler 480 (Roche); Serum Multiplex Analysis (Eve Technologies); Envision 2104 Multilabel reader (Perkin Elmer); Illumina HiSeq sequencer (Illumina); LSRII (BD Biosciences);
Data analysis	Flowjo v9; Prism v8; Excel v16.53; RStudio v1.2.5001; r packages (DADA2 v1.16; Phyloseq v1.32.0; Phytools v0.7-20; ggplot2 v3.3.0; vegan v2.5-6; factoextra v1.0.7; DeSeq2 v1.28.1; pHeatmap v1.0.12); Salmon v1.3.0; Lightcyler 480 software v1.5.1.62 SP2; Envision Manager v1.13; FACS Diva v9.0

For manuscripts utilizing custom algorithms or software that are central to the research but not yet described in published literature, software must be made available to editors and reviewers. We strongly encourage code deposition in a community repository (e.g. GitHub). See the Nature Research guidelines for submitting code & software for further information.

Data

Policy information about availability of data

All manuscripts must include a data availability statement. This statement should provide the following information, where applicable:

- Accession codes, unique identifiers, or web links for publicly available datasets
- A list of figures that have associated raw data
- A description of any restrictions on data availability

Figure 6 - RNASeq data is provided in Geo accession #: GSE180342 https://www.ncbi.nlm.nih.gov/geo/query/acc.cgi?acc=GSE180342
Silva reference dataset (v132) used to assign taxonomy to 16S sequences can be accessed at https://www.arb-silva.de/no_cache/download/archive/release_132/
All raw data is provided in labeled excel sheets in Raw_Data.zip

Field-specific reporting

Please select the one below that is the best fit for your research. If you are not sure, read the appropriate sections before making your selection.

☒ Life sciences ☐ Behavioural & social sciences ☐ Ecological, evolutionary & environmental sciences

For a reference copy of the document with all sections, see nature.com/documents/nr-reporting-summary-flat.pdf

Life sciences study design

All studies must disclose on these points even when the disclosure is negative.

Sample size

Group sizes of 4 mice were chosen for all analysis, with statistically significant differences observed between stress and control groups at this group size. Experiments were typically performed a minimum of 3 times, with an n = 4 per group. CCAC approval requires that the minimum possible number of mice be used to obtain reliable data.

Data exclusions

Any data exclusions were done after performing a statistical outlier test using Prism v8 and are indicated in the Source data file. Figures with excluded values are as follows Figure 3d (Grubbs test); Figure 4b (Grubbs Test); Figure 4h (Grubbs Test); Figure 5h (Grubbs Test); Figure 5i (Grubbs Test); Figure 6A (Grubbs Test); Figure 6c (Grubbs Test); Supplementary Figure 5A (Grubbs Test); Supplementary Figure 5B (Grubbs Test)

Replication

Typically, critical experiments were repeated a minimum of three times with n = 4 per group (total n per group 12). In many experiments, n's were greater than 12.

Specifically - Figure 1 (independent experiment); Figure 2B (replicated twice); Figure 2C (replicated once); Figure 2D (replicated twice); Figure 2E (independent experiment); Figure 2G (independent experiment); Figure 3B (replicated twice); Figure 3C (replicated 3-4 times depending on gene); Figure 3D (naive samples - replicated four times with 2 mice per group, infected samples - replicated six times with 2 mice per group); Figure 3E (independent sample); Figure 3F (replicated three times); Figure 3G (replicated three times); Figure 3H (replicated twice); Figure 3I (replicated three times); Figure 3J (independent experiment); Figure 4B (replicated 3-4 times depending on the gene); Figure 4C (replicated twice); Figure 4D (replicated twice); Figure 4E (replicated twice); Figure 4F (replicated twice); Figure 4G (replicated twice); Figure 4H (replicated 11-12 times); Figure 4I (replicated twice); Figure 5A (control,stress - replicated four times, LPS - replicated twice); Figure 5B (control,stress - replicated four times, LPS - replicated twice); Figure 5C (replicated 5 times with 2 mice per group); Figure 5D (independent experiment), Figure 5F (replicated four times); Figure 5G (replicated four times); Figure 5H (replicated five times with two mice per group); Figure 5I (replicated five times with two mice per group); Figure 6A (replicated three times); Figure 6B (replicated 3-4 times); Figure 6C (control/stress - replicated three times, control anti-IL-22 - replicated twice, control anti-CD90 independent experiment); Figure 6D (control/stress - replicated six times, stress RU486 - replicated ten times, RU486 anti-CD90 - replicated three times, RU486 anti-IL-22 - replicated four times); Figure 6E (control/stress - replicated twice, LPS/LPS + anti-CD90 - replicated three times); Figure 7A (replicated four times); Figure 7B (independent experiment); Figure 7C/D (independent experiment); Figure 7E (replicated twice); Figure 7F (replicated 2-3 times depending on the gene); Figure 7G (independent experiment); Figure 7H (independent experiment); Figure S1 (independent experiment); Figure S2 (independent experiment); Figure S3A-D (independent experiment); Figure S4A (replicated three times); Figure S4B (replicated three times) Figure S4C (replicated 2-3 times depending on gene); Figure S4D (independent experiment); Figure S5A (replicated twice); Figure S5B (replicated four times); Figure S6 (independent experiment); Figure S7A (replicated four times); Figure S7B (independent experiment); Figure S7C (control/stress - replicated twice, stress + IL-22 - independent experiment); Figure S7D (control/stress - replicated twice, stress + IL-22 - independent experiment); Figure S7E (independent experiment); Figure S7F (independent experiment); Figure S8A/B (replicated five time with control groups of 2, stress groups of 1); Figure S8C (replicated four times with 1-2 samples).

qPCR samples/replicates were excluded for the following reasons: 1) Extracted RNA was of poor quality or too low yield to analyze. 2) cDNA failed to amplify upon qPCR analysis.
Flow cytometry samples/replicates were excluded for the following reasons: 1) Low cell yield. 2) Low cell viability.

Randomization

A comparable number of mice were randomly assigned to control or experimental groups at the time of study execution. Mice were housed identically prior to experimental use. All mice for a given experiment were from the same shipment batch of mice.

Blinding

IHC analysis was performed by a third party blinded to experimental conditions.

Reporting for specific materials, systems and methods

We require information from authors about some types of materials, experimental systems and methods used in many studies. Here, indicate whether each material, system or method listed is relevant to your study. If you are not sure if a list item applies to your research, read the appropriate section before selecting a response.

Materials & experimental systems

n/a	Involved in the study
☐	☒ Antibodies
☒	☐ Eukaryotic cell lines
☒	☐ Palaeontology and archaeology
☐	☒ Animals and other organisms
☒	☐ Human research participants
☒	☐ Clinical data
☒	☐ Dual use research of concern

Methods

n/a	Involved in the study
☒	☐ ChIP-seq
☐	☒ Flow cytometry
☒	☐ MRI-based neuroimaging

Antibodies

Antibodies used	Fc block (CD16/32; mAb; Invitrogen; MA5-18012; 1:200), anti-mouse CD90.2 (53-2.1;mAb; Invitrogen; 47-0902-82; APC eflour 780; 1:600); anti-mouse CD45 (30-F11;mAb; Invitrogen; 48-0451-82; eflour450; 1:600), anti-mouse CD45 (30-F11;mAb; Invitrogen; 45-0451-82; PerCP-Cyanine5.5 ; 1:600), Lineage exclusion markers (anti-mouse CD11b (M1/70;mAb; Invitrogen; 11-0112-82; FITC; 1:400), anti-mouse CD3 [17a2;mAb; Invitrogen; 11-0032-82; FITC; 1:400), anti-mouse TER-119 (TER-119;mAb; Invitrogen; 11-5921-82; FITC; 1:400), anti-mouse Gr1 (RB6-865;mAb; Invitrogen; 11-5931-85; FITC; 1:400), anti-mouse B220(CD45R) (RA3-6B2;mAb; Invitrogen; 11-0452-82; FITC; 1:400)], anti-mouse CD11b (M1/70; mAb; Invitrogen; 93-0112-42; eFluor605NC; 1:600), anti-mouse Glucocorticoid receptor (BuGR2; mAb; 25-6189-82; Invitrogen;PE-Cyanine7; 1:20), anti-mouse CD90.2 (53-2.1;mAb; 25-0902-81; Invitrogen; PE-Cyanine7; 1:600), anti-mouse Annexin V (mAb; 17-8007-72; Invitrogen; APC; 5 mL/test), TCRb (H57-597; mAb; Invitrogen; 78-5961-82; SuperBright780; 1:600), anti-mouse CD4 (RM4-5; mAb; Invitrogen; 69-0042-82; eFluor506; 1:400), anti-mouse CD8a (53-6.7; mAb; Invitrogen; 58-0081-80; Alexa Fluor 532; 1:400), anti-mouse rorgt (B2D; mAb; Invitrogen; 17-6981-82; APC; 1:50), anti-mouse IL-22 (poly5164;polyAb; Biolegend; 516411; PerCP-Cyanine 5.5; 5mL/test), anti-mouse EpCam (CD326) (G8.8; mAb; Invitrogen; 17-5791-82;APC;1:400), antihuman/mouse phosphoSTAT-3 (Tyr705) (LUVNKLA; mAb, Invitrogen, 25-9033-41; PE Cyanine 7; 5uL/test), mouse IgG2b kappa (isotype control;eBMG2b;Invitrogen, 25-4732-81; PE Cyanine 7).
Validation	Antibodies obtained commercially have been validated by the manufacturer for the specific use presented in this study.<br><br>Fc block (CD16/32) - Reactivity: mouse; Applications: Flow cytometry<br>APC eflour 780 anti-mouse CD90.2 - Reactivity: mouse; Applications: flow cytometry, immunoprecipitation, immunohistochemistry<br>eflour450 anti-mouse CD45 - Reactivity: mouse; Applications: flow cytometry<br>PerCP-Cyanine5.5 anti-mouse CD45 - Reactivity: mouse; Applications: flow cytometry<br>FITC Lineage exclusion markers - Reactivity: mouse; Applications: flow cytometry<br>FITC anti-mouse CD3 - Reactivity: mouse; Applications: flow cytometry<br>FITC anti-mouse TER-119 - Reactivity: mouse; Applications: flow cytometry<br>FITC anti-mouse Gr1 - Reactivity: mouse; Applications: flow cytometry<br>FITC anti-mouse B220(CD45R) - Reactivity: mouse, human; Application: flow cytometry<br>eFluor605NC anti-mouse CD11b - Reactivity: mouse; Application: flow cytometric analysis, immunoprecipitation, and immunohistochemistry<br>PE-Cyanine7 anti-mouse Glucocorticoid receptor - Reactivity: mouse; Applications: flow cytometry<br>PE-Cyanine7 anti-mouse CD90.2 - Reactivity: mouse, fish; Applications: flow cytometry, immunohistochemistry<br>APC anti-mouse Annexin V - Reactivity: mouse; Applications: flow cytometry<br>SuperBright 780 TCRb - Reactivity: mouse; Applications: flow cytometry<br>eFluor506 anti-mouse CD4 - Reactivity: mouse; Applications: flow cytometry<br>Alexa Fluor 532 anti-mouse CD8a - Reactivity: mouse; Applications: flow cytometry<br>APC anti-mouse rorgt - Reactivity: mouse; Applications: flow cytometry<br>PerCP-Cyanine 5.5 anti-mouse IL-22 - Reactivity: mouse; Applications: flow cytometry<br>APC anti-mouse EpCam (CD326) - Reactivity: mouse, human, Applications: flow cytometry, Immunohistochemistry, ICC/IF, ChIP, FN.<br>PE Cyanine 7 antihuman/mouse phosphoSTAT-3 (Tyr705) - Reactivity: mouse, human; Applications: flow cytometry<br>PE Cyanine 7 mouse IgG2b kappa - Reactivity: mouse; Applications: flow cytometry<br><br>As per the company specifications, all Invitrogen antibodies have been tested by flow cytometric analysis of mouse splenocytes. BioLegend antibodies have been quality control tested by intracellular immunofluorescent staining with flow cytometric analysis.

Animals and other organisms

Policy information about studies involving animals; ARRIVE guidelines recommended for reporting animal research

Laboratory animals	Six- to -eight-week-old male C57BL/6N mice were purchased from Charles River Laboratories (QC, CAN). Six- to -eight week old male TNFKO were originally purchased from Jackson Laboratory (ME, USA) and were bred inhouse. All mice were housed in Level 2 biohazard containment under specific pathogen-free barrier conditions and maintained on a 12 h light: 12 h dark cycle, which was temperature-controlled (21°C), 30–50% humidity.
Wild animals	No wild animals were used in the study.
Field-collected samples	No field-collected samples were used in the study.
Ethics oversight	All research was reviewed and approved by the Animal Review Ethics Board (AUP# 20-12-41, replaces 17-03-10) at McMaster University, and conducted in accordance with standards set by the Canadian

Council of Animal Care.

Note that full information on the approval of the study protocol must also be provided in the manuscript.

Flow Cytometry

Plots

Confirm that:

☒ The axis labels state the marker and fluorochrome used (e.g. CD4-FITC).

☒ The axis scales are clearly visible. Include numbers along axes only for bottom left plot of group (a 'group' is an analysis of identical markers).

☒ All plots are contour plots with outliers or pseudocolor plots.

☒ A numerical value for number of cells or percentage (with statistics) is provided.

Methodology

Sample preparation	Epithelial cell and lamina propria cell isolation - Ileum was extracted from control, stressed, and IL-22-treated, stressed mice at the time of sacrifice and placed in cold PBS. Feces was removed and samples were opened longitudinally before being cut into 1 cm segments and placed in 15 mL tubes containing 10 mL PBS. Samples were washed in PBS, placed in 5 mL of 5 mM EDTA and 2 mM DTT, and incubated at 37°C. Samples were rotated at 15 rpm in a hybridization oven for 30 mins and filtered through a 100 µM filter. Following epithelial cell isolation, cells were further digested for the isolation of lamina propria cells in 12.5 µg/mL Liberase TM (Sigma-Aldrich) and 1 mg/mL DNase I (Sigma-Aldrich) at 37°C. Control samples are comprised of 2 pooled mice and all stress samples are comprised of four pooled mice. Samples were rotated at 8 rpm in a hybridization oven for 10 mins and filtered through a 100 µM filter. Samples were enriched for mononuclear cells using a 40% Percoll gradient.
Instrument	LSRII (BD Biosciences)
Software	FACS Diva v9; Flowjo v9
Cell population abundance	EpCam+ CD45- cells (30-50%) of isolated epithelial cells; CD90+ cells (20-40% of CD45+ LP cells); CD45+CD90+[IFNg (0 -75%); IL-17A (0-60%); IL-22 (0-55%)]; pSTAT-3+ (10-30% of CD45-EpCam+)
Gating strategy	All cells --> SSC vs CD45+ --> CD45+ cells --> CD90+ --> IL-22, GR, Annexin V<br>All cells --> SSC vs CD45+ --> CD45+ cells --> CD90+ --> Lineage +/-<br>All cells --> SSC vs CD45+ --> CD45+ cells --> CD90+ --> rorgt +/- -> TCRB +/- --> Lineage +/-<br>All cells --> SSC vs CD45NEG --> EpCAM+ --> pSTAT3+

☒ Tick this box to confirm that a figure exemplifying the gating strategy is provided in the Supplementary Information.
